# Brain–heart coupling in Parkinson’s disease: from autonomic dysfunction to network physiology

**DOI:** 10.3389/fphys.2026.1974885

**Published:** 2026-10-22

**Authors:** Pasquale Pagliaro

**Affiliations:** Department of Clinical and Biological Sciences, University of Turin, Orbassano, Italy

**Keywords:** autonomic nervous system, brain–heart coupling, cardiac interoception, cardiac sympathetic denervation, heart rate variability, network physiology, neurocardiology, Parkinson’s disease

## Abstract

Cardiovascular autonomic dysfunction is a well-established feature of Parkinson’s disease (PD), yet the brain and cardiovascular system are still largely investigated as separate physiological domains. This separation may overlook an important dimension of PD pathophysiology: the dynamic communication between them. Here, we examine emerging evidence that brain–heart coupling itself is altered in PD. A focused PubMed search identified studies directly relating neural activity to cardiac dynamics, complemented by relevant physiological and autonomic literature. Despite a small and heterogeneous evidence base, electrophysiological, neuroimaging, and dynamic network studies suggest disrupted central–cardiac coordination. Alterations are detectable in early and *de novo* PD, relate to dysautonomia, and appear sensitive to dopaminergic state. Emerging evidence for impaired cardiac interoception further suggests that dysfunction may extend from descending autonomic control to ascending heart-to-brain processing. Importantly, cross-system measures can reveal abnormalities that remain not captured by neural or cardiovascular signals alone. Their clinical potential is promising but remains to be established through larger, standardized, and longitudinal studies. At present, brain–heart coupling is best viewed as a physiological framework with emerging translational relevance rather than as a validated biomarker. By revealing interactions that remain hidden when neural and cardiovascular systems are examined separately, brain–heart coupling may uncover a distinct dimension of PD pathophysiology and provide a bridge from integrative physiology to disease phenotyping and future clinical stratification.

## Introduction

Parkinson’s disease (PD) is increasingly recognized as a multisystem neurodegenerative disorder rather than a predominantly motor disease. Its clinical spectrum includes numerous non-motor manifestations, with autonomic dysfunction representing a major component. Cardiovascular involvement commonly includes orthostatic hypotension (OH), baroreflex impairment, blood pressure instability, and altered heart rate variability (HRV). Although reduced HRV is the predominant group-level finding in idiopathic PD, its direction and magnitude vary across clinical and genetic subtypes. These abnormalities may occur at different disease stages and reflect dysfunction across several levels of cardiovascular control (Kaufmann and Goldstein, 2013; Jain and Goldstein, 2012). Cardiac sympathetic denervation is another characteristic feature of PD and may evolve partly independently of motor impairment and dopaminergic treatment (Goldstein et al., 2000). Clinical and experimental evidence increasingly places peripheral autonomic alterations within PD pathophysiology itself (Cuenca-Bermejo et al., 2021; Sharabi et al., 2021). These abnormalities may precede motor deficits and should not be considered merely consequences of advanced neurological disability. Instead, cardiovascular autonomic abnormalities may provide insight into disease mechanisms extending beyond the nigrostriatal motor system. Recent work has similarly highlighted cardiovascular autonomic failure across the PD continuum (Fanciulli et al., 2026).

Cardiovascular autonomic involvement in PD has traditionally been investigated using peripheral physiological and imaging measures. These include HRV, blood pressure responses, baroreflex sensitivity, autonomic function tests, and cardiac sympathetic imaging (Jain and Goldstein, 2012; Kumar et al., 2026). Such approaches have provided fundamental insights into autonomic dysfunction but cannot characterize the dynamic relationship between central neural activity and cardiovascular physiology. This limitation is particularly relevant because cardiac autonomic regulation depends on a distributed central autonomic network. This network encompasses brainstem, hypothalamic, limbic, and cortical regions involved in cardiovascular regulation. Evidence for its involvement in PD comes from studies combining central and peripheral physiological measures. In *de novo* PD, simultaneous resting-state functional magnetic resonance imaging (fMRI) and dynamic HRV revealed disrupted central parasympathetic modulation (Tessa et al., 2019). Cardiovascular autonomic dysfunction may therefore involve more than peripheral cardiac denervation. It may also reflect impaired integration between central neural networks and peripheral autonomic processes.

This perspective provides the rationale for moving beyond conventional cardiovascular dysautonomia toward the broader concept of brain–heart coupling. Brain–heart coupling describes the dynamic and potentially bidirectional relationship between neural activity and cardiac physiology. It encompasses descending autonomic regulation, ascending cardiac signaling, and interoceptive processing. This perspective is situated within the broader framework of *Network Physiology*, which conceptualizes the organism as a dynamic network of interacting physiological systems. Physiological states and functions emerge not only from the behavior of individual systems but also from the topology, strength, and temporal organization of their interactions, which may reorganize across conditions and operate over multiple timescales (Bashan et al., 2012; Bartsch et al., 2015; Ivanov, 2021). Brain–heart coupling can therefore be considered a specific subsystem of this wider physiological network. In PD, its alteration may reflect a reorganization of cross-system coordination that is not captured when neural and cardiovascular signals are examined separately. Methodological approaches to brain–heart interactions have progressively moved beyond static cross-system correlations. Time-resolved models can now characterize changing relationships between neural and cardiac dynamics and, depending on their assumptions, distinguish non-directional association from putative brain-to-heart and heart-to-brain influences. These approaches include linear and nonlinear estimators, probabilistic models, and physiologically informed methods relating electroencephalographic (EEG) or fMRI dynamics to RR intervals or derived indices of cardiac autonomic modulation (Candia-Rivera et al., 2022; Valenza et al., 2025; Candia-Rivera et al., 2026). Such measures provide complementary descriptions of brain–heart interaction, but directional statistical dependence should not by itself be interpreted as proof of direct causal communication. *Information-theoretic approaches* provide a complementary framework for quantifying brain–heart interactions. Measures of information storage, shared information, and information transfer can characterize how the history of one process contributes to predicting its own dynamics or those of another process, including potentially directional relationships (Faes et al., 2014). Predictability-based frameworks can further decompose the predictability of a target cardiac or neural process into self-, causal, and interaction components, thereby separating its internal dynamics from the contributions of other processes within the brain–heart network (Faes et al., 2016). Time-varying implementations can further track transient changes without assuming that the interaction remains stationary throughout the recording (Antonacci et al., 2023). However, the resulting estimates depend on how the neural and cardiac processes are represented and on the temporal scale at which their dynamics are analyzed.

Early electrophysiological evidence revealed altered relationships between EEG oscillations and HRV indices in PD patients with autonomic dysfunction (Liou et al., 2013). Notably, frequency-domain HRV measures alone did not clearly distinguish patients from controls. Cross-system interactions may therefore reveal physiological abnormalities that remain undetected when neural or cardiovascular measures are considered separately.

Subsequent multimodal studies have strengthened this systems-level interpretation. Iñiguez and colleagues combined fMRI with cardiac recordings to investigate heart–brain synchronization in PD (Iñiguez et al., 2022). They identified reduced synchronization involving regions of the central autonomic network. Moreover, greater disruption was associated with more severe dysautonomia. Simultaneous EEG–ECG recordings have subsequently enabled brain–heart interactions to be examined with higher temporal resolution. Candia-Rivera and colleagues found reduced coupling between alpha- and gamma-band brain connectivity and cardiac sympathetic dynamics (Candia-Rivera et al., 2024b). Dopaminergic treatment partially restored this coupling, with greater recovery associated with greater motor improvement. These findings suggest that brain–heart coupling may capture clinically relevant aspects of PD physiology. Complementary methodological approaches now relate cardiac dynamics to time-varying brain network organization (Candia-Rivera et al., 2024a). Together, these studies support a shift from analyzing isolated signals toward characterizing interactions between dynamically evolving physiological systems.

This focused review examines the emerging evidence for altered brain–heart coupling in PD. Particular emphasis is placed on neurophysiological and multimodal approaches that simultaneously characterize central and cardiac dynamics. The aim is not to provide another comprehensive account of cardiovascular dysautonomia. Instead, we ask whether brain–heart interactions provide information unavailable from neural or cardiac signals considered separately. We also examine their potential contribution to systems-level physiological biomarkers of PD.

A focused PubMed search was conducted from database inception to August 14, 2026. Two complementary field-restricted searches were used. The first combined PD terms with brain–heart interaction, brain–heart coupling, heart–brain synchronization, heartbeat-evoked potential, and cardiac interoception terms. The second combined PD terms with EEG, electroencephalography, fMRI, functional magnetic resonance, brain connectivity, or cortical activity terms and with ECG, electrocardiography, HRV, or heart rate variability terms. The two searches identified a total of 32 unique records, with no overlap between them.

Reports were eligible for the focused evidence synthesis when they included patients with PD, used simultaneous or temporally aligned central and cardiac measurements, and explicitly assessed their relationship. Studies reporting neural and cardiac variables only as separate outcomes, studies addressing exclusively peripheral cardiovascular dysfunction, and studies in which PD was mentioned only within a broader neurological context were excluded. Six reports met these criteria, and one additional eligible report was identified through reference-list screening. The resulting seven reports are summarized in **Table 1**. Because the two reports by Candia-Rivera and colleagues analyzed overlapping PD data, they represent six independent patient cohorts. Additional publications were included to provide the broader physiological, clinical, and methodological context of the review, but were not considered part of the direct PD evidence synthesis.

**Table 1:** Main studies directly assessing brain–heart interactions in Parkinson’s disease.

Study/population	Neural and cardiac signals	Coupling approach	Main finding/clinical relevance	Principal limitation
Liou et al. (2013) 26 PD with autonomic dysfunction; 23 controls	Resting EEG; ECG-derived frequency-domain HRV	Regional EEG spectral power ↔ HRV	Cortical–autonomic associations differed between groups despite limited discrimination by HRV alone	Cross-sectional; correlational; no directionality; PD cohort selected for autonomic dysfunction
Tessa et al. (2019) 14 *de novo*, drug-naïve PD; 14 controls	Resting-state fMRI BOLD; dynamic HF-HRV	Spontaneous HF-HRV ↔ regional BOLD activity	Networks associated with parasympathetic cardiac modulation were disrupted in early untreated PD	Small cohort; limited BOLD temporal resolution; predominantly parasympathetic cardiac index
Beach and Miocinovic (2021) 7 early PD; 10 controls; PD examined OFF/ON levodopa	64-channel EEG; ECG-derived HRV	Heartbeat-evoked potentials	Preliminary evidence of altered heart-to-brain processing; HEP and HRV shifted toward control values ON levodopa	Very small sample; conference abstract; methodological sensitivity of HEPs
Iñiguez et al. (2022) 31 PD; 21 controls	Resting-state fMRI BOLD; photoplethysmography-derived HRV	Lagged HRV–BOLD cross-correlation; HBSI	Heart–brain synchronization was reduced in CAN-related regions and associated with dysautonomia	Cross-sectional; synchronization does not establish directionality; cardiac signal derived from photoplethysmography
Candia-Rivera et al. (2024b) 15 PD OFF/ON medication; 16 controls	Resting EEG dynamic connectivity; ECG-derived autonomic dynamics	Brain-connectivity fluctuations ↔ cardiac dynamics	Alpha/gamma–sympathetic coupling was reduced OFF medication and partly restored ON; recovery correlated with motor improvement	Proof-of-concept; small cohort; directionality unresolved; medication effects potentially multifactorial
Candia-Rivera et al. (2024a) Overlapping PD dataset plus independent healthy dataset	Dynamic EEG network topology; ECG-derived sympathetic–vagal dynamics	Cardiac dynamics ↔ clustering, efficiency, modularity, and assortativity	Network-level coupling provided information about physiological and medication state	Methodological proof-of-concept; PD dataset not an independent replication cohort
Pardo-Rodriguez et al. (2021); PD and healthy controls	EEG and ECG-derived cardiac dynamics	Cortical–vagal network analysis	Preliminary evidence of disrupted cortical–vagal communication in PD	Conference proceedings; small exploratory study; limited methodological and clinical detail

BOLD, blood-oxygen-level-dependent; CAN, central autonomic network; ECG, electrocardiography; EEG, electroencephalography; fMRI, functional magnetic resonance imaging; HBSI, heart–brain synchronization index; HEP, heartbeat-evoked potential; HF, high frequency; HRV, heart rate variability; PD, Parkinson’s disease.

The two Candia-Rivera, 2024 studies include overlapping PD data and should not be considered independent replication cohorts.

### Physiological basis of brain–heart interactions

Brain–heart interactions depend on continuous bidirectional communication across a distributed neurocardiac axis. The heart therefore represents more than a passive target of central autonomic commands. At the central level, cardiovascular regulation is coordinated by the central autonomic network (CAN). This distributed network integrates visceral information with cognitive, emotional, behavioral, and homeostatic demands. Its major components include the insular and cingulate cortices, amygdala, hypothalamus, and periaqueductal gray. Brainstem structures include the parabrachial complex, nucleus of the solitary tract (NTS), and ventrolateral medulla (Benarroch, 1993; Mazzola et al., 2023). Importantly, these central structures do not operate in isolation. They interact continuously with spinal, intrathoracic, and intracardiac neuronal networks. Cardiac neural control therefore emerges from nested feedback loops operating across multiple levels of the neuraxis. These loops extend from higher brain centers to the intrinsic cardiac nervous system (ICNS) (**Figure 1**) (Ardell et al., 2016; Ardell and Armour, 2016). This organization extends cardiac autonomic control beyond a purely top-down hierarchical model toward an integrated network of reciprocal interactions. Such a systems perspective also supports the emerging concept of a bidirectional brain–heart axis in neurodegenerative disease (Sian-Hulsmann and Riederer, 2026). This framework is consistent with the *neurovisceral integration model*, which links cardiac vagal regulation to the functional integrity of a hierarchically organized network of prefrontal, limbic, hypothalamic, and brainstem structures (Thayer and Lane, 2000, 2009; Smith et al., 2017). Applied to PD, this framework encompasses central autonomic network dysfunction, sympathetic and parasympathetic efferent pathways, processing within intrathoracic and intrinsic cardiac neural networks, and vagal and spinal afferent feedback to the brain. These components form recurrent regulatory loops, although the extent to which each is affected may differ across patients and PD subtypes.

**Figure 1:**
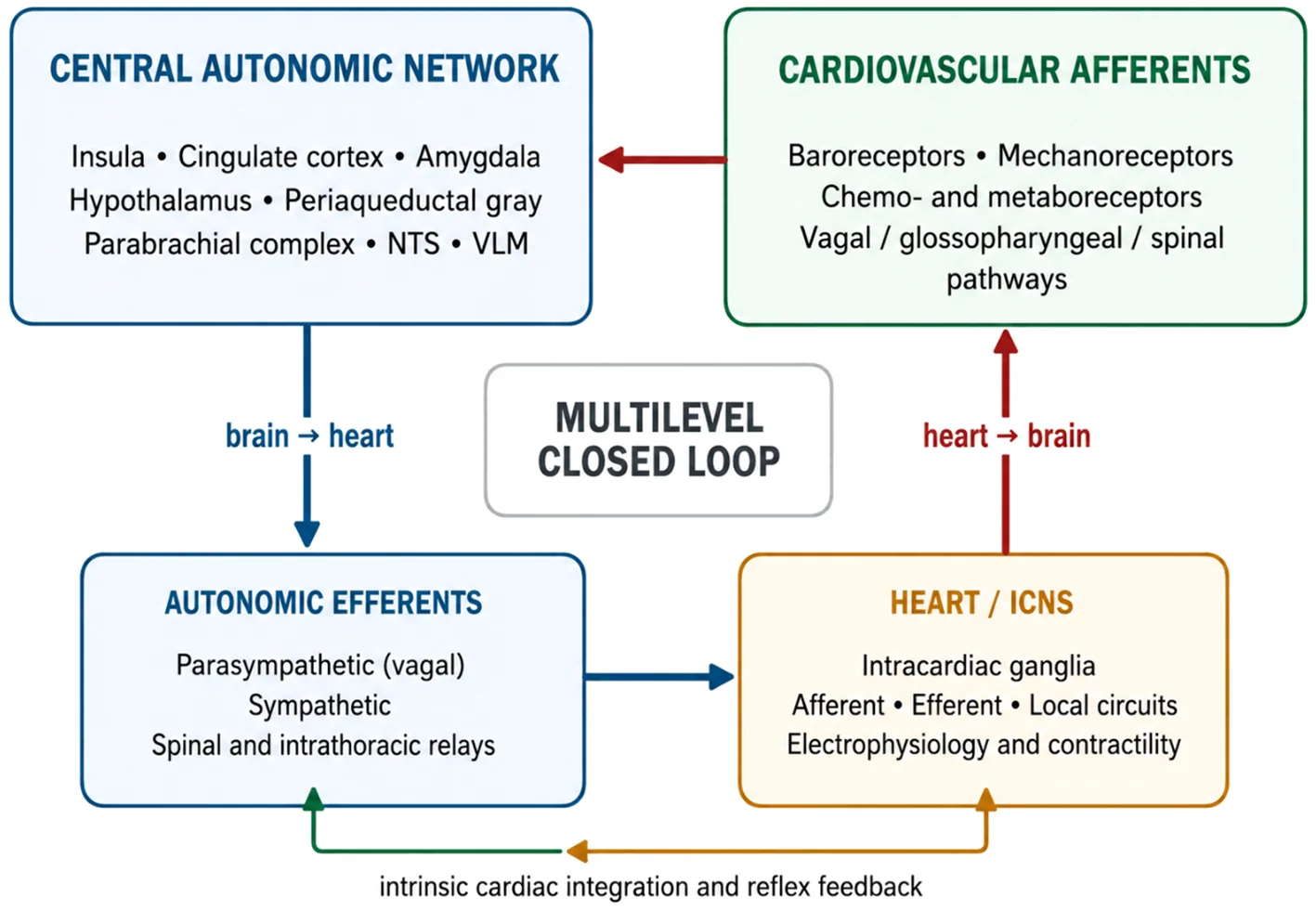
Multilevel organization of bidirectional brain–heart communication. The CAN modulates cardiac function through parasympathetic and sympathetic efferent pathways. Cardiovascular afferents convey information from the heart and circulation to central autonomic structures. These signals arise from baroreceptors, mechanoreceptors, chemoreceptors, and metaboreceptors and travel through vagal, glossopharyngeal, and spinal pathways. The ICNS provides an additional level of neural integration. Cardiac regulation therefore emerges from recurrent interactions among central, extracardiac, and intracardiac neural circuits. CAN, central autonomic network; ICNS, intrinsic cardiac nervous system; NTS, nucleus of the solitary tract; PAG, periaqueductal gray; VLM, ventrolateral medulla.

The efferent, or brain-to-heart, arm of this system operates primarily through sympathetic and parasympathetic pathways. Cardiac sympathetic preganglionic neurons originate mainly within the thoracic spinal cord and project to paravertebral and intrathoracic ganglia. Postganglionic cardiac projections then modulate chronotropy, dromotropy, inotropy, lusitropy, and electrophysiological properties. Parasympathetic cardiac control is predominantly mediated through the vagus nerve. Preganglionic neurons arise from medullary nuclei and synapse with postganglionic neurons located within or near the heart. Vagal regulation is classically associated with rapid control of sinoatrial and atrioventricular nodal activity. However, its influence extends beyond the atria and cardiac conduction system. Anatomical, neurochemical, and physiological evidence supports substantial parasympathetic innervation of the ventricular myocardium (Coote, 2013). Vagal activation can also modulate ventricular refractoriness, action potential duration, contractility, and rhythm (Coote, 2013). Sympathetic and parasympathetic influences should therefore not be viewed as independent or simply antagonistic. Their combined effects are often non-additive and depend on the prevailing activity of each autonomic branch. A classic example is “accentuated antagonism,” whereby vagal effects can become stronger during concurrent sympathetic activation (Coote, 2013). This interaction may involve both prejunctional modulation of neurotransmitter release and postjunctional signaling within cardiac cells. However, accentuated antagonism is not observed under all experimental conditions, and its determinants remain incompletely understood (Elawa et al., 2022).

Importantly, extrinsic autonomic pathways do not act on the myocardium in isolation. The heart contains an extensive intrinsic cardiac nervous system (ICNS), organized predominantly within ganglionated plexuses. This network comprises afferent neurons, efferent neurons, and local circuit interneurons. Its functional complexity has led to its description as the cardiac “little brain” (Armour, 2008). Intrinsic cardiac neurons receive information about the local mechanical and chemical environment. They integrate these signals with extracardiac autonomic inputs and contribute to regional cardiac regulation. The ICNS therefore represents more than a final parasympathetic relay. It functions as a local integrative network that modifies autonomic inputs before they reach myocardial targets (Armour, 2008; Ardell and Armour, 2016). This organization supports regulation across multiple timescales, from beat-to-beat adjustments to longer-term neurocardiac adaptations. Cardiac function therefore emerges from interactions among central, intrathoracic, intracardiac, and myocardial components (Ardell et al., 2016). This distributed organization challenges the concept of cardiac regulation as the output of a single descending autonomic command.

The same framework also requires a careful interpretation of heart rate variability (HRV). Beat-to-beat fluctuations in cardiac cycle length reflect autonomic modulation of sinoatrial activity. However, they are also shaped by respiration, baroreflex mechanisms, sympathetic and vagal dynamics, and their interactions. HRV therefore provides an informative peripheral readout of cardiac autonomic regulation. It does not directly measure sympathetic or parasympathetic neural firing (Candia-Rivera et al., 2026). Nor should HRV itself be considered a measure of brain–heart coupling. Similar cardiac dynamics may arise from different combinations of central output, peripheral processing, reflex activity, and intrinsic cardiac integration. Thus, comparable HRV patterns do not necessarily imply comparable underlying neurocardiac physiology. This distinction becomes particularly relevant in neurological disorders, where central–peripheral autonomic relationships may themselves be altered.

Crucially, communication along the neurocardiac axis is not exclusively descending. The heart continuously generates sensory information about its mechanical, hemodynamic, and chemical state. This information reflects myocardial deformation, chamber pressure, and conditions within the coronary and great-vessel circulation. Cardiac sensory signals reach the central nervous system through vagal and spinal afferent pathways. Arterial baroreceptor signals are conveyed primarily through vagal and glossopharyngeal afferents. The NTS represents a major integration site for these visceral inputs. It interacts with other brainstem nuclei and with hypothalamic, limbic, and cortical structures (van Weperen and Vaseghi, 2023; Ardell et al., 2016). Cardiac afferent signals therefore contribute to reflex cardiovascular regulation while also reaching higher-order neural systems. These systems participate in arousal, emotion, cognition, and perception.

Cardiac afferent signaling is therefore more than a feedback mechanism for correcting cardiovascular disturbances. Afferent and efferent pathways form recurrent loops that continuously adapt cardiovascular function to physiological demands (Fukuda et al., 2015). Within this framework, each heartbeat is partly the consequence of preceding neural and hemodynamic events. Yet each heartbeat also generates new sensory information capable of influencing subsequent neural activity. This cyclical organization provides a physiological substrate for measurable brain–heart coupling. Neural activity can be related to subsequent changes in cardiac dynamics. Conversely, heartbeat timing and cardiac phase can be related to ongoing cortical oscillations and network activity (Candia-Rivera et al., 2021, 2026). Brain-to-heart and heart-to-brain influences should therefore be viewed as interacting components of a continuous physiological loop.

Ascending cardiac signaling also provides the physiological basis for cardiac interoception. Cardiac interoception involves sensing, integrating, and representing the internal cardiovascular state. It extends beyond conscious heartbeat perception and includes processing of cardiovascular afferent information across multiple neural levels. These include brainstem, subcortical, and cortical structures. The insular cortex has a particularly important role in this process. It contributes both to representing internal bodily states and to descending autonomic regulation (Benarroch, 1993; Nagai et al., 2026). The insula therefore provides a functional interface between heart-to-brain and brain-to-heart signaling. Experimentally, the cortical processing of cardiac signals can be investigated using heartbeat-evoked potentials or responses (HEPs/HERs), obtained by averaging EEG or magnetoencephalographic (MEG) activity time-locked to individual cardiac events. These signals are commonly interpreted as markers of heart-to-brain processing, but they are not direct measures of cardiac afferent integrity. Their amplitude depends on interoceptive attention, respiratory phase, behavioral state, and analytical choices, including cardiac-event definition, EEG reference, artifact correction, and the selected time window. In healthy participants, cardiac interoceptive attention enhances late HEP responses predominantly during exhalation, while respiratory phase also modulates heartbeat-related cortical oscillations and connectivity (Zaccaro et al., 2024, 2026). HEP findings in PD should therefore be interpreted in relation to respiratory and attentional conditions and to the analytical pipeline used. Other methods assess cardiac phase-dependent modulation of neural oscillations and functional connectivity (Candia-Rivera et al., 2021, 2026).

Taken together, these observations support a multilevel closed-loop model of brain–heart interaction. This system encompasses central autonomic networks, spinal and intrathoracic pathways, autonomic efferents, the ICNS, and ascending visceral afferents. The myocardium and vasculature are also integral components of this functional network. Dysfunction at one level may therefore influence the behavior of the entire system. This framework is particularly relevant to PD, where central and peripheral autonomic abnormalities may coexist. Neurodegeneration may involve central autonomic structures alongside cardiac sympathetic denervation and other peripheral abnormalities (Jain and Goldstein, 2012; Martello and Lionetti, 2026). The relevant pathophysiological unit may therefore be the neurocardiac system rather than either component considered in isolation. From this perspective, brain–heart dysfunction may reflect altered integration across multiple levels of the neurocardiac axis.

### Cardiovascular autonomic dysfunction in Parkinson’s disease

Cardiovascular autonomic dysfunction is among the best-established non-motor manifestations of PD, although its expression varies across patients and disease stages. Abnormalities include changes in HRV, impaired cardiovagal and sympathoneural baroreflex responses, OH, altered circadian blood pressure regulation, chronotropic incompetence, and cardiac sympathetic denervation. Reduced HRV is the predominant finding in idiopathic PD (Jain and Goldstein, 2012; Martello and Lionetti, 2026), although this pattern is not shared by all genetic subtypes (Carricarte Naranjo et al., 2019, 2025). Importantly, these abnormalities should not be regarded as equivalent manifestations of a single autonomic lesion. Rather, they probe distinct components of cardiovascular control, from dynamic sinoatrial modulation to reflex integration and peripheral sympathetic integrity. Their partial dissociation is particularly relevant to brain–heart coupling. It suggests that cardiovascular autonomic dysfunction in PD involves multiple levels of the neurocardiac axis.

#### Heart rate variability and cardiac autonomic modulation

HRV has been extensively used to characterize cardiac autonomic dysfunction in PD. It provides a non-invasive measure of beat-to-beat variability in sinus rhythm. Reductions have been reported in the standard deviation of normal-to-normal intervals (SDNN) and the root mean square of successive differences (RMSSD) (Heimrich et al., 2021). SDNN reflects overall variability of normal-to-normal intervals, whereas RMSSD predominantly captures short-term vagally mediated beat-to-beat variability. Abnormalities have also been reported in frequency-domain measures, particularly high-frequency (HF) power, consistent with impaired vagal modulation. However, findings vary with recording duration, respiratory control, posture, medication state, disease severity, age, and analytical methodology. A meta-analysis of 47 studies involving 2,772 participants found significantly reduced HF power and RMSSD in PD (Heimrich et al., 2021). Heterogeneity was substantial, and evidence of publication bias was present for HF power. Short-term RMSSD, particularly under controlled respiration, emerged as one of the more robust markers of impaired parasympathetic modulation. This predominant pattern should not, however, be interpreted as a universal feature of PD. Genotype-related differences have been reported, most notably in PD associated with the LRRK2 G2019S variant. Patients with LRRK2-PD showed increased beat-to-beat variability, deceleration capacity, and heart-rate irregularity compared with controls or patients with idiopathic PD, suggesting a distinct autonomic phenotype (Carricarte Naranjo et al., 2019). More recently, a similar HRV profile was identified in a subgroup of non-manifesting LRRK2 G2019S carriers who also displayed greater motor and non-motor involvement, raising the possibility that increased HRV may precede conversion to clinically manifest PD in a subset of carriers (Carricarte Naranjo et al., 2025). These findings indicate that reduced HRV represents a prevalent group-level feature of predominantly idiopathic PD cohorts rather than a uniform disease signature. They also reinforce the need to consider genotype and clinical phenotype when interpreting HRV in PD. Consistent with this heterogeneity, a recent small study found preserved deceleration capacity in PD, whereas marked reductions were observed in multiple system atrophy and progressive supranuclear palsy (Ruppert et al., 2025).

The physiological interpretation of HRV nevertheless requires caution. Low-frequency (LF) HRV should not be considered a direct quantitative measure of cardiac sympathetic activity. Likewise, the LF/HF ratio should not be interpreted uncritically as “sympathovagal balance.” HRV reflects integrated influences from autonomic, respiratory, baroreflex, hemodynamic, and intrinsic cardiac mechanisms. It therefore describes modulation of sinoatrial rhythm rather than the firing activity of a single autonomic branch. This distinction is especially important in PD, where both central and peripheral autonomic pathways may be affected.

Recent physiological studies reinforce this interpretation. Cernanova Krohova and colleagues combined conventional HRV with causal baroreflex and information-theoretic analyses in early PD (Cernanova Krohova et al., 2025). During orthostatic stress, patients showed reduced HF-HRV together with lower causal baroreflex sensitivity. Abnormalities were particularly evident in patients with concomitant gastrointestinal autonomic features. Similarly, PD patients studied during OFF and ON dopaminergic states showed reduced SDNN and impaired responses to deep breathing (Kumar et al., 2026). Baroreflex sensitivity was also reduced relative to controls. Together, these findings illustrate why HRV becomes more informative when integrated with physiological challenges and complementary measures of reflex control. Nevertheless, HRV abnormalities identify altered cardiac autonomic modulation but cannot, by themselves, localize its origin along the neurocardiac axis. Moreover, the direction of these abnormalities may differ across PD subtypes, as illustrated by the increased HRV reported in LRRK2 G2019S-associated PD (Carricarte Naranjo et al., 2019).

#### Baroreflex dysfunction

The arterial baroreflex is particularly relevant because it is an inherently closed-loop cardiovascular control mechanism. It links peripheral hemodynamic changes to central integration and autonomic efferent responses. A fall in arterial pressure unloads arterial baroreceptors and reduces afferent signaling to the NTS. The resulting response combines cardiac vagal withdrawal with increased sympathetic outflow to the heart and vasculature. These adjustments increase heart rate, cardiac performance, vascular resistance, and venous return. An increase in arterial pressure elicits the opposite response. Effective orthostatic regulation therefore requires coordinated afferent sensing, brainstem integration, cardiovagal modulation, and sympathoneural output. In healthy subjects, engagement of both cardiac and sympathetic baroreflex components increases with orthostatic challenge and is not determined simply by the absolute value or direction of arterial pressure changes (De Maria et al., 2023).

In PD, dysfunction may occur at several points within this reflex arc. Cardiovagal baroreflex sensitivity and sympathoneural responses to falling pressure may both be attenuated, particularly in neurogenic orthostatic hypotension (nOH) (Kaufmann and Goldstein, 2013). “Baroreflex failure” should therefore not be regarded as a unitary phenomenon. Reflex control of heart period and sympathetic vasoconstrictor output represent related but partly separable physiological processes (Kaufmann and Goldstein, 2013; De Maria et al., 2023). Reduced baroreflex sensitivity has also been demonstrated using standardized cardiovascular autonomic testing (Kumar et al., 2026). More advanced analyses suggest that respiratory–cardiac and baroreflex interactions may already be altered in very early PD (Cernanova Krohova et al., 2025).

Baroreflex impairment provides a mechanistic link between altered autonomic modulation and clinically evident blood pressure instability. Standing reduces central blood volume and requires rapid sympathetic vasoconstriction and appropriate cardiac compensation. Failure of these responses favors a sustained fall in arterial pressure. The effect may become particularly important when impaired reflex control coexists with peripheral noradrenergic denervation. Thus, orthostatic cardiovascular dysfunction in PD can emerge from abnormalities distributed across the reflex loop rather than from a single lesion.

#### Orthostatic hypotension and neurogenic circulatory failure

Orthostatic hypotension is the most clinically evident manifestation of cardiovascular autonomic dysfunction in PD. A recent meta-analysis of 7,748 individuals estimated an overall prevalence of approximately 33% (Wang et al., 2025). However, prevalence varies substantially across studies. Age, disease duration, medication, hydration, comorbidities, testing protocols, and diagnostic criteria all contribute to this variability. Distinguishing nOH from non-neurogenic OH is therefore essential.

In nOH, falling arterial pressure elicits an inappropriately small compensatory increase in heart rate. This response reflects impaired autonomic reflex activation. The relationship between orthostatic heart-rate and systolic-pressure changes can therefore help identify a neurogenic mechanism. Longitudinal data also demonstrate the importance of disease stage. Among 907 participants with early PD, nOH was present in only 3.9% at baseline (Beach and McKay, 2024). Its prevalence increased over four years, and affected patients showed greater motor and functional impairment. This estimate is considerably lower than the approximately one-third prevalence reported across broader PD populations (Wang et al., 2025). The difference is informative rather than contradictory. It highlights the effects of disease stage, cohort selection, and physiological definitions on prevalence estimates.

The pathophysiology of nOH involves inadequate sympathetic noradrenergic activation during standing. Both baroreflex dysfunction and peripheral sympathetic denervation may contribute. Vascular resistance and venous return consequently fail to increase sufficiently as central blood volume falls. Some patients also develop supine hypertension, illustrating the broader instability of short-term blood pressure control. Dopaminergic and other medications can further modify orthostatic responses. However, nOH cannot be regarded simply as a pharmacological consequence. Cardiovascular autonomic dysfunction occurs in untreated and early PD and may precede advanced motor disease (Jain and Goldstein, 2012; Kumar et al., 2026).

#### Cardiac sympathetic denervation: a peripheral hallmark of PD

Postganglionic cardiac sympathetic denervation is one of the most characteristic cardiovascular autonomic abnormalities in PD. Early neurochemical and imaging studies demonstrated substantial loss of myocardial sympathetic innervation (Goldstein et al., 2000). This loss is reflected by reduced myocardial uptake of ^123I-metaiodobenzylguanidine (MIBG), a norepinephrine analog handled by sympathetic nerve terminals. Importantly, reduced MIBG uptake can occur relatively early in PD. Its severity does not necessarily parallel nigrostriatal dopaminergic dysfunction or orthostatic blood pressure abnormalities.

Neuropathological evidence supports these imaging observations. Loss of tyrosine-hydroxylase-positive sympathetic fibers has been demonstrated in cardiac tissue from individuals with incidental Lewy body disease (Orimo et al., 2007). This condition is considered a possible presymptomatic stage within the Lewy-body disease spectrum. In some cases, cardiac sympathetic fiber loss preceded detectable neuronal loss in major central autonomic structures. Peripheral cardiac autonomic pathology may therefore emerge early rather than simply reflecting advanced central neurodegeneration. This interpretation is consistent with broader evidence that autonomic innervation of the heart, gut, and skin may be affected by α-synuclein pathology early in PD (Sharabi et al., 2021). However, the temporal relationship between peripheral and central pathology remains heterogeneous and incompletely resolved.

Imaging studies also reveal substantial heterogeneity. A review encompassing 54 studies and 3,114 individuals with PD evaluated the diagnostic performance of cardiac MIBG scintigraphy (Pitton Rissardo and Fornari Caprara, 2023). Reported sensitivity was approximately 0.81–0.83 and specificity approximately 0.80–0.86, depending on acquisition phase. Reduced cardiac MIBG uptake is therefore highly characteristic but not universal in PD. Preserved cardiac sympathetic innervation in some patients may reflect meaningful biological heterogeneity rather than merely diagnostic imperfection.

Longitudinal observations strengthen this interpretation. Yoo and colleagues followed 122 patients with early drug-naïve PD using repeated myocardial MIBG imaging (Yoo et al., 2024). Over approximately four to five years, distinct trajectories of cardiac sympathetic involvement emerged. Some patients showed denervation at baseline, others developed it during follow-up, and some retained relatively preserved innervation. These trajectories paralleled aspects of central neurobiology and may therefore identify biologically distinct PD phenotypes. In another cohort, reduced MIBG uptake was associated with greater overall PD burden after accounting for orthostatic pressure changes (Yoo et al., 2021). Cardiac sympathetic denervation and OH are therefore related manifestations, but they should not be considered interchangeable.

A possible relationship with α-synuclein pathology adds another dimension. In early PD, plasma α-synuclein levels correlated with myocardial MIBG uptake independently of striatal dopamine transporter binding (Park et al., 2022). This observation requires independent replication and does not establish causality. Nevertheless, it raises the possibility that peripheral cardiac involvement reflects disease processes not captured by conventional measures of nigrostriatal degeneration.

#### From autonomic phenotype to brain–heart dysfunction

Taken together, HRV abnormalities, baroreflex impairment, nOH, and cardiac sympathetic denervation establish cardiovascular dysautonomia as an integral component of PD. However, these measures interrogate different levels of the neurocardiac axis. HRV describes the temporal modulation of cardiac rhythm, whereas baroreflex testing examines closed-loop reflex control. OH represents the hemodynamic consequence of inadequate cardiovascular compensation. MIBG imaging instead assesses the integrity of postganglionic cardiac sympathetic terminals. These measures are complementary rather than interchangeable. Their incomplete correspondence explains why no single conventional autonomic marker can characterize cardiovascular neural control in PD.

This heterogeneity provides the physiological rationale for studying brain–heart coupling directly. Central autonomic regulation, reflex integration, peripheral innervation, and cardiac responses may become partly dissociated during PD progression. Measurements confined to either the brain or cardiovascular periphery may therefore miss changes in their functional relationship. The relevant question is no longer simply whether cardiovascular autonomic dysfunction occurs in PD; this is well established. The emerging question is whether and how PD also disrupts the dynamic coordination through which brain and cardiovascular physiology operate as an integrated system.

### Direct neurophysiological evidence of altered brain–heart coupling in Parkinson’s disease

Cardiovascular dysautonomia in PD does not, by itself, demonstrate altered communication between the brain and heart. Evidence for brain–heart coupling dysfunction requires simultaneous or temporally aligned measurements of central neural and cardiac dynamics. Their statistical or physiological interdependence must then be explicitly assessed. This distinction is important because neural and cardiac abnormalities may coexist without implying disruption of their functional relationship. Nevertheless, studies using EEG–ECG, fMRI–HRV, heartbeat-evoked responses, and dynamic network approaches provide convergent evidence of altered central–cardiac coordination in PD.

One of the earliest electrophysiological observations was reported by Liou and colleagues (Liou et al., 2013). They simultaneously recorded EEG and ECG in 26 patients with PD and autonomic dysfunction and 23 matched controls. Spectral EEG analysis was combined with frequency-domain HRV to examine relationships between cortical oscillations and cardiac autonomic modulation. Importantly, conventional HRV measures did not clearly distinguish patients from controls despite greater autonomic symptom burden in PD. By contrast, cortical–autonomic relationships differed between groups. Controls showed specific associations between autonomic indices and activity within lateral premotor–parietal circuits. These relationships were reorganized in PD. Thus, altered cross-system relationships were detectable even when conventional cardiac measures alone showed limited group discrimination. However, the analysis was correlation-based and provided no information about directionality or time-varying coupling. The study therefore demonstrated altered central–autonomic association rather than bidirectional brain–heart communication.

A complementary perspective was provided by Tessa and colleagues in 14 *de novo*, untreated patients and 14 controls (Tessa et al., 2019). Resting-state fMRI was combined with time-varying estimates of high-frequency HRV. In controls, spontaneous HF-HRV fluctuations were associated with blood-oxygen-level-dependent (BOLD) activity across distributed cortical, subcortical, and brainstem regions. This pattern included structures belonging to the central autonomic network. In PD, this coordinated pattern was substantially disrupted. Differences involved the cingulate and insular cortices, prefrontal and sensorimotor regions, supplementary motor area, hippocampus, and precuneus. Importantly, patients were drug-naïve and at an early clinical stage. The findings therefore cannot readily be attributed to chronic dopaminergic treatment or advanced disability. They suggest that altered central modulation of cardiac parasympathetic dynamics may emerge early in PD.

Iñiguez and colleagues subsequently examined heart–brain synchronization in 31 patients with PD and 21 matched controls (Iñiguez et al., 2022). Resting-state BOLD activity was recorded synchronously with cardiac signals derived from photoplethysmography. Time-varying HRV was then related to regional brain activity using lagged cross-correlation analysis. The authors derived a heart–brain synchronization index (HBSI) quantifying synchronization between HRV fluctuations and cerebral BOLD dynamics. HBSI was reduced in PD across several regions related to the central autonomic network. Importantly, greater desynchronization was associated with more severe dysautonomia. HBSI also correlated with objective measures of sympathetic and cardiovagal function. These included Valsalva pressure recovery and heart-rate responses during deep breathing. Brain–heart synchronization therefore showed not only disease-related alterations but also clinically meaningful physiological associations.

A different approach addresses the ascending, heart-to-brain component through heartbeat-evoked potentials (HEPs). Beach and Miocinovic recorded simultaneous 64-channel EEG and ECG in seven patients with early PD and ten controls (Beach and Miocinovic, 2021). Patients were examined in relation to levodopa exposure. HEPs represent cortical activity time-locked to cardiac events and provide a neurophysiological measure of heartbeat-related cortical processing. HEP amplitude was altered in the OFF-medication state, together with changes in HRV. Levodopa shifted both measures toward control values. These preliminary observations suggest that PD may affect the cerebral processing of cardiac information, not only descending autonomic regulation. However, the study was very small and reported only as a conference abstract. HEPs also present methodological challenges, including cardiac field artefacts and sensitivity to EEG referencing and preprocessing (Candia-Rivera et al., 2021). Evidence for altered heart-to-brain processing in PD should therefore remain provisional.

A major methodological advance came from analyzing dynamic interactions between whole-brain connectivity and cardiac autonomic fluctuations. Candia-Rivera and colleagues used simultaneous resting-state EEG and ECG to quantify these interactions (Candia-Rivera et al., 2024b). The study included 15 patients examined both ON and OFF dopaminergic medication and 16 healthy controls. EEG functional connectivity was characterized across canonical frequency bands. Beat-to-beat cardiac dynamics were decomposed into faster predominantly vagal and slower sympathetic-related components. The analysis then quantified how changes in brain connectivity evolved together with changes in cardiac autonomic dynamics.

The strongest abnormalities involved cardiac sympathetic dynamics and cortical connectivity within the alpha and gamma frequency ranges. Patients studied OFF medication showed reduced coupling compared with healthy controls. Crucially, the abnormality emerged at the level of cross-system coordination rather than either signal considered independently. This finding shifts the physiological focus from abnormalities within individual systems toward altered interactions between them.

The ON–OFF comparison also provided an informative physiological perturbation. Dopaminergic treatment partially restored abnormal brain–heart coupling (Candia-Rivera et al., 2024b). Greater coupling recovery was associated with greater motor improvement. These observations suggest that brain–heart coordination may be sensitive to dopaminergic state. However, they do not establish a direct dopaminergic mechanism. Levodopa simultaneously affects basal ganglia activity, cortical oscillations, movement, arousal, blood pressure, and peripheral autonomic physiology. Medication state should therefore be considered both a potential confounder and an experimental probe. Future studies should determine whether coupling changes track dopamine replacement, motor improvement, autonomic effects, dyskinesia, or their interaction.

The same dataset was subsequently used to examine coupling between cardiac dynamics and time-varying brain network topology (Candia-Rivera et al., 2024a). EEG connectivity was characterized using clustering, global efficiency, modularity, and assortativity across alpha, beta, and gamma bands. These network properties were related to dynamically estimated cardiac sympathetic–vagal activity. This approach represents the brain as an evolving network rather than a single oscillator or isolated cortical region. Brain–heart coupling consequently becomes an interaction between two complex physiological systems changing over time. In PD, abnormalities particularly involved relationships between cardiac dynamics and brain network segregation. Coupling measures also provided information about medication state beyond individual brain or cardiac features. These analyses remain proof-of-concept and require independent replication. Nevertheless, they provide a framework for conceptualizing PD as a disorder of network physiology extending beyond the brain.

Taken together, these studies reveal a coherent pattern despite substantial methodological heterogeneity. Altered brain–heart relationships have been detected using EEG oscillations, EEG functional connectivity, and BOLD activity. The phenomenon therefore does not appear restricted to a single recording modality. Abnormalities have also been observed in early and *de novo* PD (Tessa et al., 2019). Altered coupling is therefore unlikely to represent exclusively a late consequence of severe autonomic failure. Moreover, coupling measures have been associated with dysautonomia severity and dopaminergic motor response (Iñiguez et al., 2022; Candia-Rivera et al., 2024b). Some studies also suggest that cross-system measures provide information not captured by neural or cardiac indices alone (Liou et al., 2013; Candia-Rivera et al., 2024a) (**Figure 2**). The evidence remains limited, but its convergence supports altered brain–heart coordination as a distinct physiological dimension of PD.

**Figure 2:**
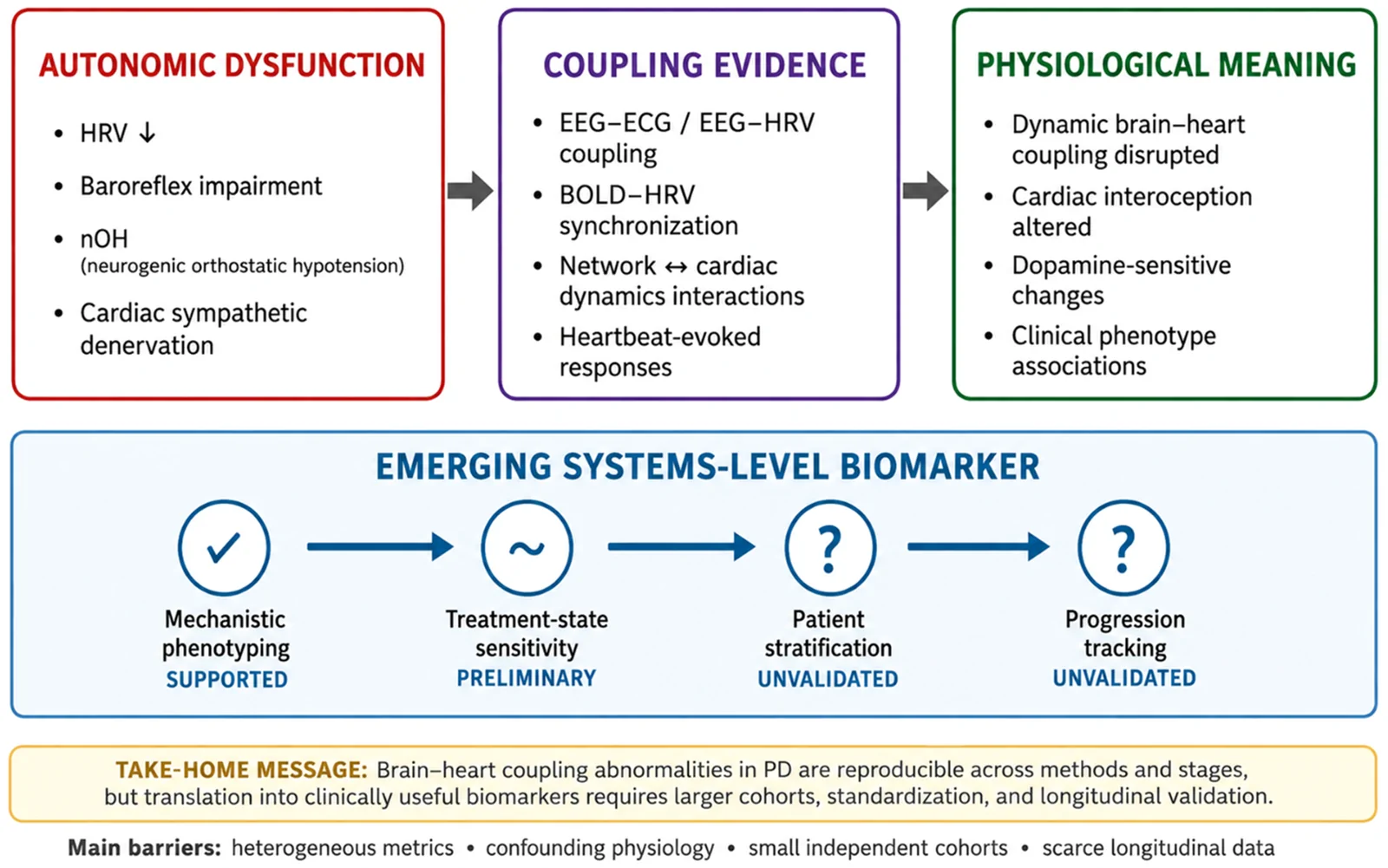
Conceptual synthesis of brain–heart dysfunction in Parkinson’s disease. Established cardiovascular autonomic abnormalities define the conventional autonomic phenotype of PD. EEG–ECG, fMRI–HRV, dynamic network analyses, and heartbeat-evoked approaches provide emerging evidence of altered cross-system coordination. Together, these findings support brain–heart coupling as a candidate systems-level physiological biomarker. However, its application to patient stratification and disease progression remains unvalidated. Major barriers include methodological heterogeneity, physiological confounding, small independent cohorts, and limited longitudinal data. BOLD, blood-oxygen-level-dependent; ECG, electrocardiography; EEG, electroencephalography; fMRI, functional magnetic resonance imaging; HRV, heart rate variability; nOH, neurogenic orthostatic hypotension; PD, Parkinson’s disease.

Despite this convergence, the evidence remains preliminary. Most studies involve small samples and cross-sectional designs, while definitions of brain–heart coupling vary considerably. Some analyses also rely on overlapping datasets, further reducing the number of independent PD cohorts. These limitations constrain generalizability and prevent firm conclusions about directionality or clinical utility. They do not, however, eliminate the consistent physiological evidence of altered central–cardiac coordination.

The current evidence therefore supports a physiological rather than diagnostic interpretation. PD appears to affect the dynamic relationship between central neural activity and cardiac autonomic regulation. This relationship may provide information beyond abnormalities detected within either system alone. **Table 1** summarizes the available direct evidence, its clinical relevance, and its main methodological limitations. The following sections examine the complementary heart-to-brain component and the potential translational value of these cross-system measures.

### Heart-to-brain signaling and interoception

Most studies of cardiovascular autonomic dysfunction in PD have adopted a predominantly *brain-to-heart* perspective. They have focused on how neurodegeneration affects autonomic control of cardiovascular function. However, brain–heart communication is intrinsically bidirectional. Each cardiac cycle generates mechanical, hemodynamic, and chemical information that reaches the brain through visceral afferent pathways. These signals contribute to homeostatic regulation and to the neural representation of the internal bodily state. Altered *heart-to-brain* signaling may therefore represent a complementary component of brain–heart dysfunction in PD.

Interoception in PD has recently been comprehensively reviewed, including its neuroanatomical substrates, clinical manifestations, and translational implications (Longardner et al., 2025). Here, we address a narrower question directly relevant to brain–heart coupling: whether PD alters the processing of cardiac afferent information. Cardiac afferent processing forms part of interoception, the sensing, integration, and interpretation of signals originating within the body. Interoception extends well beyond conscious awareness of visceral events. It ranges from largely unconscious homeostatic processing to cortical representation and conscious perception of bodily states. These processes integrate bottom-up visceral signals with attention, affective state, prior experience, and top-down expectations. Cardiac interoception specifically concerns the processing and representation of cardiovascular signals, including those generated by individual heartbeats. This body–brain interface has recently emerged as a potentially important but understudied dimension of PD (Longardner et al., 2025).

Several features of PD make altered interoception biologically plausible. Neurodegeneration can involve structures participating in visceral processing and autonomic integration. These include brainstem nuclei, the locus coeruleus, hypothalamic and limbic structures, and the insular and cingulate cortices. At the same time, peripheral autonomic pathology may alter the visceral information reaching the central nervous system. Interoceptive dysfunction could therefore arise at several levels. These include altered peripheral signaling, impaired central representation, or abnormal integration of afferent information with higher-order processes (Longardner et al., 2025). In PD, altered interoception may therefore reflect dysfunction of the entire afferent–central processing pathway rather than a purely sensory deficit.

Initial behavioral evidence supports altered cardiac interoception in PD. Ricciardi and colleagues assessed interoceptive sensitivity using a heartbeat-perception task (Ricciardi et al., 2016). Patients with PD were less accurate than healthy controls in perceiving their own heartbeats. This deficit was not clearly related to motor severity or several non-motor and affective symptoms. Cardiac interoceptive impairment may therefore not simply parallel conventional measures of disease severity. However, heartbeat-counting tasks require cautious interpretation. Performance can depend on attention, time estimation, beliefs, cognitive strategies, and prior knowledge of heart rate. Such tasks therefore provide indirect measures of cardiac interoception rather than pure measures of afferent signal fidelity.

The heterogeneity of interoceptive impairment was further illustrated by Santangelo and colleagues (Santangelo et al., 2018). They compared heartbeat perception and subjective interoceptive sensibility across different PD phenotypes and healthy controls. Patients with tremor-dominant PD showed reduced interoceptive accuracy and sensibility compared with both other groups. Their performance on a time-estimation control task was preserved, arguing against a nonspecific timing deficit. In contrast, subjective interoceptive sensibility in the postural instability/gait difficulty phenotype was associated with several affective and behavioral features. These included anxiety, anhedonia, and impulse-control disturbances. Interoceptive dysfunction may therefore vary across PD phenotypes rather than simply increasing with overall disease severity.

This possibility has clinically relevant implications. Objective autonomic dysfunction and subjective autonomic awareness may become dissociated in PD. Patients can exhibit physiologically important abnormalities without reporting symptoms of comparable severity. Orthostatic hypotension provides a relevant example, because impaired awareness of falling blood pressure may delay compensatory behavior. Similar mismatches may occur in other visceral domains. Longardner and colleagues proposed that impaired interoception could contribute to such “silent” autonomic disturbances (Longardner et al., 2025). However, direct longitudinal evidence linking impaired interoception to clinical outcomes remains limited.

An intriguing extension of this concept concerns the perception of myocardial ischemia. Ischemic cardiac pain depends partly on intact visceral afferent transmission and central nociceptive processing (Di Franco et al., 2015). Pain processing is also altered in PD, although experimental evidence generally indicates increased pain sensitivity, particularly OFF medication (Thompson et al., 2017). Whether PD modifies the perception of myocardial ischemia remains unknown. This question deserves investigation, particularly when cardiac autonomic denervation and impaired interoception coexist.

Behavioral measures cannot establish where altered heartbeat perception originates. Reduced performance may reflect abnormal cardiac afferent signaling, altered cortical processing, or higher-order cognitive factors. HEPs provide a more direct neurophysiological approach. HEPs are EEG responses obtained by averaging cortical activity time-locked to cardiac events, usually the ECG R wave. They provide an electrophysiological measure of heartbeat-related cortical processing. However, HEPs should not be interpreted as direct recordings of a single cardiac afferent pathway. Their amplitude and topography depend on afferent signaling, attention, arousal, cortical state, and methodological choices (Candia-Rivera et al., 2021).

Direct HEP evidence in PD remains extremely limited. Beach and Miocinovic recorded simultaneous 64-channel EEG and ECG in seven patients with early PD and ten controls (Beach and Miocinovic, 2021). Patients were examined in relation to levodopa state. HEP amplitude was altered OFF medication, together with reduced HRV. Levodopa shifted both HEP and cardiac measures toward control values. These observations provide preliminary evidence that PD may alter cortical processing of heartbeat-related information. They also suggest possible sensitivity to dopaminergic state. However, the sample was very small and the findings were reported only as a conference abstract. The study should therefore be considered hypothesis-generating rather than confirmatory evidence of altered cardiac afferent processing.

The apparent levodopa sensitivity of HEPs is intriguing but mechanistically ambiguous. Dopaminergic treatment simultaneously affects cortical state, attention, autonomic outflow, and cardiac physiology. Apparent HEP normalization therefore cannot be attributed specifically to restored cardiac afferent processing. Dynamic EEG–ECG coupling also shows partial recovery during dopaminergic treatment (Candia-Rivera et al., 2024b). Together, these observations suggest more broadly that dopaminergic state may influence central–peripheral physiological integration.

Methodological limitations further constrain interpretation of HEP findings. A recent meta-analysis found substantial heterogeneity in latency windows, scalp topographies, referencing schemes, and analytical procedures (Wilkinson et al., 2026). Across clinical populations, no consistent overall difference in HEP amplitude from healthy controls was demonstrated. Effect estimates were relatively large in neurological and cardiovascular conditions but were based on few studies. HEP methodology therefore remains insufficiently standardized for clinical biomarker use. This problem is particularly relevant in PD. Cardiac field artefacts, tremor, movement, medication, cognition, respiration, autonomic dysfunction, and cardiovascular comorbidities may all influence HEP measurements.

A critical distinction should therefore be maintained. Evidence for interoceptive dysfunction in PD is stronger than evidence for specifically impaired cardiac afferent processing. Behavioral studies indicate impaired representation of cardiac bodily states in at least some patients (Ricciardi et al., 2016; Santangelo et al., 2018). Clinical observations also suggest mismatches between objective autonomic dysfunction and subjective awareness (Longardner et al., 2025). By contrast, direct electrophysiological evidence remains largely confined to preliminary HEP findings (Beach and Miocinovic, 2021). Behavioral evidence of impaired cardiac interoception should therefore not be interpreted as direct evidence of altered cardiac afferent processing.

Nevertheless, the heart-to-brain perspective adds an important dimension to brain–heart dysfunction in PD. Peripheral cardiovascular pathology may alter the signals reaching the brain. Neurodegeneration may simultaneously affect the networks responsible for processing those signals. Their interaction could therefore disrupt closed-loop integration rather than simply autonomic output or visceral perception alone. The heart becomes both a target of neural regulation and a continuous source of information shaping central activity. Whether disruption of this recurrent loop contributes to autonomic unawareness, non-motor symptoms, or PD heterogeneity remains an important open question.

### Translational potential of brain–heart coupling

Altered brain–heart interactions in PD raise a broader translational question: can cross-system physiological measures provide information that conventional neurological and autonomic assessments miss? PD affects multiple levels of the neurocardiac axis, whereas most conventional measurements characterize individual components of this system. Brain–heart measures instead quantify the functional relationship between neural and cardiovascular dynamics. Their potential value therefore lies first in physiological characterization and only subsequently in biomarker development. Current evidence supports biological plausibility, disease association, clinical correlates, and sensitivity to physiological state. Evidence for reproducibility, longitudinal validity, individual-level classification, and clinically meaningful thresholds remains limited.

A candidate biomarker should first provide information beyond conventional measurements. Early EEG–ECG findings are relevant in this respect. Liou and colleagues identified altered cortical–autonomic relationships despite limited group discrimination by frequency-domain HRV alone (Liou et al., 2013). More recent network approaches similarly suggest that dynamic brain–heart coupling may provide information beyond either system considered separately (Candia-Rivera et al., 2024a). This potential incremental information represents a major rationale for developing brain–heart measures as physiological biomarkers. Whether they improve prediction beyond simpler measures remains to be established.

Clinical relevance provides a second requirement. Reduced heart–brain synchronization has been associated with greater autonomic dysfunction in PD (Iñiguez et al., 2022). Brain–heart coupling may therefore reflect the functional consequences of autonomic network disruption rather than simply distinguish patients from controls. However, its relationship with the broader non-motor phenotype remains unclear. Cognition, sleep, mood, fatigue, gastrointestinal dysfunction, and other manifestations of PD have received limited investigation in this context. This question is particularly relevant because interoceptive abnormalities themselves appear heterogeneous across PD phenotypes (Santangelo et al., 2018).

Brain–heart measures may also be sensitive to motor and dopaminergic state. Candia-Rivera and colleagues found reduced coupling between cardiac sympathetic dynamics and alpha- and gamma-band brain connectivity OFF medication (Candia-Rivera et al., 2024b). Dopaminergic treatment partially restored this coupling, and greater recovery was associated with greater motor improvement. Preliminary HEP observations similarly suggest medication-related changes in cortical responses to cardiac signals (Beach and Miocinovic, 2021). Brain–heart coupling may therefore contain both trait-like and state-sensitive components.

State sensitivity is potentially valuable but complicates interpretation. An ON–OFF difference cannot be assumed to reflect direct dopaminergic normalization of brain–heart physiology. Levodopa simultaneously modifies basal ganglia activity, cortical oscillations, motor performance, arousal, blood pressure, and peripheral autonomic function. Moreover, dopaminergic treatment can produce biologically distinct therapeutic and dyskinetic effects (Bordbar et al., 2026). Future studies should therefore distinguish medication state from motor response, autonomic effects, and dyskinesia. Dopaminergic treatment should therefore be considered both a potential confounder and a useful physiological perturbation for investigating brain–heart interactions.

Whether brain–heart coupling can track disease progression is even less certain. Conventional autonomic abnormalities clearly evolve during PD. Neurogenic OH becomes more prevalent longitudinally (Beach and McKay, 2024), while serial MIBG imaging reveals heterogeneous trajectories of sympathetic denervation (Yoo et al., 2024). Brain–heart relationships might therefore also change as the disease progresses. However, this hypothesis remains essentially untested. Direct coupling studies are predominantly cross-sectional, and within-subject longitudinal trajectories have not been established. Brain–heart coupling cannot therefore currently be considered a progression biomarker.

A related possibility is its use for PD phenotyping. Cardiac sympathetic denervation, OH, interoceptive impairment, and central network dysfunction do not necessarily evolve in parallel. Cross-system measures might therefore identify patients with different patterns of central–peripheral integration. This possibility is consistent with heterogeneity in cardiac interoception and peripheral sympathetic involvement (Santangelo et al., 2018; Yoo et al., 2024). However, no brain–heart coupling metric has been prospectively validated for patient stratification.

Deep brain stimulation (DBS) offers a particularly interesting experimental model. Basal ganglia stimulation can modify cardiovascular autonomic function, although reported effects vary across targets and stimulation parameters (Cucinotta et al., 2024). DBS provides a controlled perturbation of central networks with potential downstream autonomic consequences. However, evidence that DBS directly reorganizes brain–heart coupling is currently lacking. Simultaneous neural and cardiovascular recordings during DBS could help test causal relationships between central network activity and cardiac dynamics.

Plantar mechanical stimulation provides a complementary peripheral perturbational approach. In a small study of patients with PD, controlled stimulation of foot sensory afferents improved gait and enhanced cardiac and vascular sympathetic modulation during standing 24 h later (Barbic et al., 2014). Model-free causality analysis of the same intervention also detected increased systolic arterial pressure-to-heart period coupling, consistent with improved baroreflex involvement (Bassani et al., 2014). These findings suggest that afferent mechanical stimulation may provide both an experimental probe and a potential device-based intervention for autonomic dysfunction in PD, although confirmation in larger controlled studies is required.

Several methodological requirements must be addressed before clinical translation:

First, greater standardization is needed because a brain–heart coupling measure depends not only on the underlying physiology but also on how neural and cardiac dynamics are represented. Neural activity may be described by EEG amplitude, oscillatory power, functional connectivity, BOLD fluctuations, or network topology, whereas cardiac dynamics may be represented by RR intervals, conventional HRV indices, or model-derived autonomic components. These choices, together with window length, sampling procedure, temporal resolution, frequency range, and coupling estimator, can substantially influence the detected interaction (Candia-Rivera et al., 2026). This is particularly important because neural and cardiovascular dynamics operate across different characteristic time scales, and the presence and strength of their coupling may change with the scale of observation (Ivanov, 2021; Vergara et al., 2025). Future studies should therefore report these analytical choices explicitly, examine robustness across physiologically relevant time scales, distinguish brain-to-heart, heart-to-brain, and non-directional coupling, and validate these constructs against independent physiological measures.

Second, potential confounders should be treated as physiological variables rather than residual noise. Respiration is particularly important because it influences HRV, blood pressure, cortical activity, and brain oscillations. However, it is not merely a trivial variable: respiratory phase can actively modulate the cortical processing of cardiac signals. In healthy participants, exhalation enhanced heartbeat-evoked potentials during cardiac interoceptive attention and modulated heartbeat-related cortical oscillations and functional connectivity (Zaccaro et al., 2024, 2026). Respiration may therefore contribute to genuine cardio-respiratory–brain coordination while also generating or modifying apparent brain–heart coupling. It should ideally be recorded simultaneously and explicitly modeled in terms of respiratory phase, rate, and depth. Posture, vigilance, movement, tremor, age, cardiovascular disease, physical fitness, and medications can also affect both systems. PD introduces additional variability through motor fluctuations, dyskinesia, and autonomic medications.

Third, electrophysiological approaches present specific technical challenges. The cardiac electrical field can contaminate scalp EEG, particularly during heartbeat-evoked analyses. HEP amplitude and topography also depend on EEG referencing and preprocessing choices (Candia-Rivera et al., 2021). More fundamentally, statistical dependence between neural and cardiac signals does not establish direct communication or directionality. Common inputs, including respiration and blood pressure fluctuations, may generate apparent coupling.

Finally, the current evidence base remains small. Most studies include only a few tens of participants, and some analyses rely on overlapping datasets. Clinical validation will require independent replication, test–retest reliability, standardized acquisition, and robustness across analytical pipelines. Candidate measures must also demonstrate incremental value over simpler physiological markers.

Group-level differences alone are insufficient for clinical use. The eventual goal would be an individual-level measure with a clear physiological interpretation. Such measures might distinguish impaired descending control, altered cardiac afferent processing, or disrupted bidirectional integration. Multimodal recordings combining EEG, ECG, continuous blood pressure, respiration, and conventional autonomic testing may help achieve this goal. However, additional complexity is justified only when it improves physiological stratification or clinical prediction.

Brain–heart coupling therefore remains a candidate physiological biomarker rather than a validated clinical biomarker. Its biological plausibility is strong, and disease association is supported by several independent methodological approaches. Clinical relevance is suggested by relationships with dysautonomia and motor response. Preliminary ON–OFF observations also support sensitivity to physiological state. In contrast, reproducibility, longitudinal validity, prognostic value, and individual-level discrimination remain largely unestablished.

Its immediate value may therefore lie less in diagnosing PD than in characterizing its systems-level physiology. Brain–heart measures could reveal aspects of disease organization that neural or cardiovascular measurements cannot capture separately. Their greatest potential may be to quantify how effectively neural and cardiovascular networks remain dynamically integrated across disease states and therapeutic interventions.

## Discussion and future directions

Conventional cardiovascular autonomic assessment remains essential for characterizing dysautonomia in PD. The four cardiovascular autonomic reflex tests (CARTs) include heart-rate response to deep breathing, the 30:15 ratio during active standing, the Valsalva maneuver, and the blood-pressure response to standing. These non-invasive, standardized, and reproducible tests have age-adjusted normative values and represent the clinical gold standard for assessing cardiovascular autonomic function and diagnosing cardiovascular autonomic neuropathy (Spallone et al., 2011). In PD, CART likewise represents the reference method for confirming autonomic failure (Vallelonga et al., 2021). Tilt-table testing, resting or spectral HRV, baroreflex analysis, cardiac sympathetic imaging, and tests of other autonomic domains, such as quantitative sudomotor axon reflex testing (QSART), provide complementary physiological information.

The current evidence supports a shift from viewing cardiovascular autonomic dysfunction as an isolated non-motor feature of PD toward considering altered neurocardiac integration as part of disease physiology. The relevant pathophysiological unit may therefore extend beyond either the brain or heart considered in isolation. It may encompass their continuous interaction through central networks, autonomic pathways, intrinsic cardiac circuitry, and ascending visceral signals.

Current evidence supports this framework but remains insufficient to define a specific brain–heart signature of PD. Altered central–cardiac relationships have been detected using electrophysiological and neuroimaging approaches, including in early disease. Some measures also correlate with dysautonomia and appear sensitive to dopaminergic state. However, the heart-to-brain component remains considerably less explored. Behavioral studies support altered cardiac interoception, whereas direct electrophysiological evidence of abnormal cardiac afferent processing remains preliminary. What remains unclear is how brain–heart communication is altered across different stages and phenotypes of PD, and whether the disturbance primarily involves descending, ascending, or bidirectional pathways.

Answering this question will require a transition from small cross-sectional studies to hypothesis-driven longitudinal physiology. Future studies should combine neural recordings with ECG, continuous blood pressure, respiration, and conventional cardiovascular autonomic phenotyping. Here, conventional phenotyping refers to the assessment of autonomic symptoms together with established measures such as resting HRV, heart-rate and blood-pressure responses to active standing or head-up tilt, deep breathing and Valsalva maneuver, baroreflex sensitivity, and, when available, cardiac sympathetic imaging (Jain and Goldstein, 2012; Kumar et al., 2026). Brain–heart coupling analysis can use simultaneously or temporally aligned neural and cardiac signals to quantify their dynamic statistical or physiological interdependence. Depending on the analytical method, it may assess non-directional coordination, directed brain-to-heart influences, or heart-to-brain interactions related to afferent cardiac signaling. It should therefore be viewed as complementary to, rather than a replacement for, conventional autonomic phenotyping. Importantly, statistical coupling does not by itself demonstrate direct communication or causality, because the observed association may also arise from shared influences such as respiration, blood pressure, arousal, or movement. Nevertheless, brain-to-heart, heart-to-brain, and shared coupling should be distinguished whenever possible. When clear-cut PD subtypes are unavailable, tool selection should be driven by the physiological question rather than by a presumed subtype. EEG–ECG combined with continuous blood-pressure and respiratory recordings may be particularly suitable for temporal and directional analyses, whereas fMRI–ECG may provide better anatomical localization of central network correlates. Coupling measures should be interpreted alongside CARTs and after accounting for genotype, disease stage, medication state, and autonomic symptom burden, as multidimensional physiological profiles rather than as evidence of discrete subtypes.

Future studies should also move beyond resting-state characterization and actively perturb the neurocardiac loop. Orthostatic stress, dopaminergic manipulation, controlled neuromodulation, and sensory reduction could test how coupling changes when the system is challenged. Such paradigms may help distinguish impaired afferent signaling, altered central integration, and abnormal autonomic output. Sensory-reduced environments, including anechoic conditions, are particularly interesting because they could test whether reducing exteroceptive competition modifies cardiac interoception or heart-to-brain coupling. Longitudinal studies should then establish whether coupling abnormalities precede, accompany, or follow clinically evident autonomic dysfunction.

The significance of this approach extends beyond the search for another diagnostic biomarker. Brain–heart coupling provides a means of probing network physiology across organ systems. It may reveal disease heterogeneity that remains invisible when neural and cardiovascular signals are analyzed separately. Genetic forms of PD illustrate how different central and peripheral substrates might generate distinct neurocardiac phenotypes. LRRK2-PD has been associated with greater basal forebrain volume, increased cortical and thalamic acetylcholinesterase activity, and increased HRV, consistent with a potentially preserved or compensatory cholinergic phenotype (Liu et al., 2018; Batzu et al., 2023; Carricarte Naranjo et al., 2019). In contrast, idiopathic and GBA-PD show reduced HRV, with GBA-PD exhibiting greater cardiovascular autonomic impairment (Carandina et al., 2022). Because these findings derive from separate studies rather than simultaneous brain–heart measurements, they represent candidate substrates rather than established coupling phenotypes. *The brain-first/body-first framework* offers a further, although hypothetical, interpretation of neurocardiac heterogeneity. Early peripheral autonomic involvement in body-first PD and initially greater preservation of peripheral autonomic structures in brain-first PD might generate different temporal patterns of descending and ascending brain–heart dysfunction (Borghammer and Van Den Berge, 2019; Horsager et al., 2020). This possibility has not been directly tested using brain–heart coupling measures. Moreover, the framework is not a strict binary classification: subtype markers remain imperfect, trajectories may converge with disease progression, and mechanisms other than α-synuclein spread may influence neurocardiac function (Horsager and Borghammer, 2024).

Reproducible coupling phenotypes, integrating central and peripheral measurements within the same individuals, could eventually contribute to physiological and pathophysiological stratification, progression monitoring, or assessment of therapeutic responses. These applications remain to be demonstrated. For now, the strongest contribution of brain–heart coupling is conceptual and mechanistic. PD may involve not only dysfunction within the brain and autonomic cardiovascular system, but also disruption of the dynamic relationships that integrate them.
